# Lambdoid synostosis: endoscopic craniectomy

**DOI:** 10.3171/2021.1.FOCVID20131

**Published:** 2021-04-01

**Authors:** David F. Jimenez

**Affiliations:** Pediatric Neurosurgery, El Paso Children's Hospital, El Paso, Texas

**Keywords:** endoscopy, craniosynostosis, craniectomy, osteotomy, lambdoid

## Abstract

Lambdoid craniosynostosis leads to significant deformational changes of the calvaria and cranial fossae. Surgery used to treat the condition typically consists of a calvarial vault remodeling (CVR) procedure whereby the entire occiput is removed and reshaped along with a bandeau advancement to give the patient a rounded occiput. As an option, this video presents the minimally invasive endoscopic craniectomy used at the author's institution, which was developed there and has been successfully used for 25 years. This procedure is simple and can be done rapidly, with minimal to no blood loss. The video details the key steps necessary to successfully perform the procedure.

The video can be found here: https://vimeo.com/515746378.

## Transcript

Lambdoid craniosynostosis often leads to significant and complex cranial and facial deformities, as seen in these two infants. To make the correct diagnosis, computer tomograms are not only necessary but mandatory. Surgical treatment typically calls for some type of posterior calvarial vault remodel surgery.

**0:49 Surgical Technique**. Presented in this video is our technique for performing endoscopic release of the lambdoid suture. The patient is placed in the lateral decubitus position.

**1:00 Incisions**. Surgical access to the stenosed lambdoid suture is done with two relatively small incisions that are strategically located. The first one is over the lambda, as shown here. A bloodless opening is made with the monopolar needle tip on cut mode set at 15 W. The second incision is made medial to the affected asterion, as shown here. Dissection is carefully taken down to the deeper levels to reach the subgaleal plane. This plane is then developed with the aid of a Bovie needle tip.

**1:28 Galeal Dissection**. The subgaleal plane between the two incisions is carefully and bloodlessly developed with the aid of a rhinoplasty lighted retractor. A longer Bovie tip is then necessary to reach the middle of the dissection field. The rhinoplasty lighted retractor, seen in the surgeon's left hand, is very useful in developing this critical surgical plane. The fully developed subgaleal plane is now inspected with the aid of a 0° 4-mm rigid endoscope.

**2:13 Burr Hole**. A pediatric perforator is used to create a burr hole at the lambda incision. It is then elongated with Kerrisons, to allow the insertion of a 30° endoscope aimed at the undersurface of the bone. As the endoscope is advanced, a Frazier sucker is then moved in tandem to separate the dura from the overlying bone. The lambdoid suture is replaced by a bony ridge indenting the dura, as seen here. At the level of the asterion, one can see the open suture and its invaginating dural fibers. Another burr hole is then placed next to the open suture at the level of the asterion. This burr hole is then enlarged with Kerrison rongeurs, toward the lambda in preparation for the osteotomy.

**3:17 Osteotomy**. Now that the bone has been freed from the scalp above and from the dura below, a linear craniectomy can be made with a pair of Mayo scissors or bone-cutting scissors. Two osteotomies are sequentially made. There the posterior cut is made, carefully connecting both burr holes. The scissors are then turned so that the anterior osteotomy can now be made. Care is taken to aim the tip of the scissors upward as to minimize the chances for an unintended durotomy. The osteotomy need not be very large and should measure about 5 mm or so. The cut bone can then be easily removed through one of the incisions. The completed craniectomy can be inspected with the aid of an endoscope.

**4:10 Osseous Coagulation**. In order to minimize chances of a postoperative hematoma, coagulation of the diploic spaces is easily and rapidly done with a suction coagulator, which is then connected to a Bovie unit and set at 60 W on the coagulation mode. The scalp and the dura are protected with insulated coated brain ribbons.

Postoperative pain is primarily controlled by injection of bupivacaine with epinephrine at 1 ml/kg applied to both incisions as well as a greater occipital nerve block.

**4:35 Wound Closure**. The galea is then closed with 4-0 interrupted Monocryl absorbable sutures, closing both incisions simultaneously and rapidly. The dermis is then closed with Steri-Strips and Mastisol.

This simple and elegant technique can lead to significant correction of skull base abnormalities, as shown in this 3-month-old male 11 months after the surgery. Calvarial deformities are also corrected, as seen in this 4-month-old female at 7 months and then at 2 years following endoscopic suturectomy.

**5:44 Conclusion**. Thus, in conclusion, endoscopic-assisted craniectomies for the treatment of lambdoid synostosis in infants is a safe and effective surgical option for the treatment of this problem. It is associated with minimal blood loss, no blood transfusions, minimal pain, and overall excellent outcomes.

**5:59 References** ^1–4^

## Acknowledgments

I would like to acknowledge Constance M. Barone, MD, for her key role in developing and refining the technique.
